# Prediction, Knowledge, and Explainability: Examining the Use of General Value Functions in Machine Knowledge

**DOI:** 10.3389/frai.2022.826724

**Published:** 2022-03-31

**Authors:** Alex Kearney, Johannes Günther, Patrick M. Pilarski

**Affiliations:** ^1^Reinforcement Learning and Artificial Intelligence Lab, Department of Computing Science, University of Alberta, Edmonton, AB, Canada; ^2^Alberta Machine Intelligence Institute, Edmonton, AB, Canada; ^3^Department of Medicine, University of Alberta, Edmonton, AB, Canada

**Keywords:** reinforcement learning, General Value Functions, knowledge, prediction, explainability

## Abstract

Within computational reinforcement learning, a growing body of work seeks to express an agent's knowledge of its world through large collections of predictions. While systems that encode predictions as General Value Functions (GVFs) have seen numerous developments in both theory and application, whether such approaches are explainable is unexplored. In this perspective piece, we explore GVFs as a form of explainable AI. To do so, we articulate a subjective agent-centric approach to explainability in sequential decision-making tasks. We propose that prior to explaining its decisions to others, an self-supervised agent must be able to introspectively explain decisions to itself. To clarify this point, we review prior applications of GVFs that involve human-agent collaboration. In doing so, we demonstrate that by making their subjective explanations public, predictive knowledge agents can improve the clarity of their operation in collaborative tasks.

## 1. A Subjective View on Agents

It is advantageous for agents to learn about the world that they are operating in. An agent deployed for a sufficient amount of time will face circumstances unforeseen by its creator, making the ability adapt and react to new experiences essential. Computational reinforcement learning (RL) methods that adapt based on experience have had numerous recent successes in both research domains and applied settings (Mnih et al., 2015; Silver et al., 2017; Lazic et al., 2018; Bellemare et al., 2020); however, how agents make decisions is often opaque, leading to concerns about how agents will perform in new circumstances (Adadi and Berrada, 2018; Holzinger, 2018). In this article, we concern ourselves with how artificial agents in sequential decision-making problems can explain their decisions. In particular, we focus on systems that model their world by learning large collections of predictions encoded as General Value Functions (GVFs) (White et al., 2015), an area of Machine Intelligence in which explainability has yet to be explored. To do so, we argue for a subjective, and self-supervised approach to explainability.

## 2. Predictions: The Foundation of Agent Perception

Evidence suggests that the best ways for an agent to construct an independent conception of the world is through their subjective experiences (Brooks, 2019). One way for agents to learn about their experience is to model their world as many interrelated predictions (or *forecasts*) (White et al., 2015). These predictions can be as simple as “If I keep walking forwards, will I bump into something?”, and can be so complex as to map out the entirety of the environment an agent exists in Ring (2021).

By learning predictions, an agent's conception of the world can be composed exclusively of interrelations of sensations and actions over time. Forecasts can be made and learned in an entirely self-supervised fashion: agents do not require labeled examples nor a human ontology to form abstract relationships. This enables agents to construct new abstract relationships from its own stream of experience by adding new predictions over time (Sherstan et al., 2018b; Veeriah et al., 2019; Kearney et al., 2021) without human prompting or input.

Understanding perception through the lens of prediction has a basis not only in AI, but also in biological systems, such as humans (Wolpert et al., 1995; Rao and Ballard, 1999). Humans are constantly making predictions about what they expect to see next in order to anticipate and react to their environment (Gilbert, 2009; Clark, 2015). It is no surprise then, that predictive approaches to machine perception have also been successful. Recent examples include industrial laser welding (Günther, 2018), robot navigation (Daftry et al., 2016), facilitate walking in decerebrate cats (Dalrymple et al., 2020), and control of bionic upper-limb prosthetics (Edwards et al., 2016a). A core theme that unifies this broad collection of research is (1) the ability to flexibly adapt to new situations by predicting future stimuli, and (2) learning predictions using experience.

There have been numerous advances in the algorithms underpinning predictive machine perception, and applications in real-world settings (e.g., Sherstan et al., 2015; Edwards et al., 2016a; Günther, 2018; Veeriah et al., 2019; Dalrymple et al., 2020; Schlegel et al., 2021); however, explainability of predictive knowledge systems is as of yet unexplored. In this article, we take a first look at explainability in General Value Function (GVF) systems by reflecting on recent advances in the literature. In doing so, we highlight the importance of introspection: How an agent can examine its own subjective experience and explain decisions to itself. We then focus on how the private subjective explanation can be made public, and explore examples where such explanations led to success in joint-action systems between humans and agents.

## 3. Value Functions and How They Are Estimated

**Figure 1:**
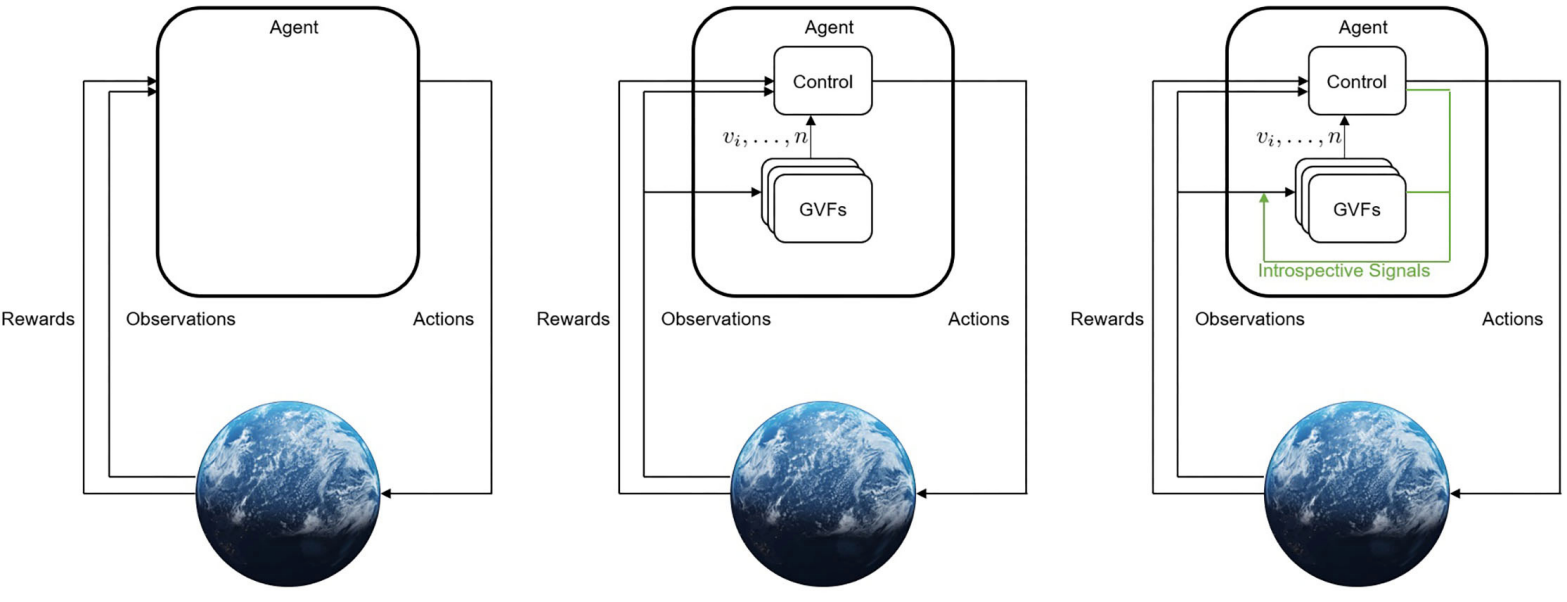
From left to right: (1) Classical RL where an agent takes actions and receives a reward and observations from its environment; (2) a setting where an agent maintains *n* GVFs about its environment to augment its observations with; (3) a setting where the agent monitors its internal learning process to further inform its estimates. Additional signals that are produced during the process of learning can be used to inform the GVFs themselves, allowing the learner access to its internal mechanisms.

To explore the explainability of predictive knowledge systems, we must first understand how they are specified and learned (Figure 1). The core component of predictive knowledge systems are their learned GVFs: forecasts of future *returns* of signals from the agent's environment. One of the best understood ways of making temporally-extended predictions is through temporal difference (TD) learning (Sutton, 1988). In temporal difference learning, time is broken down into discrete *time-steps* *t*_0_⋯*t*_*n*_. On each time-step, the agent observes the environment as *o*_*t*_: a tuple of values that include everything the agent can perceive (e.g., sensor readings, inputs from a camera). Using TD learning the *value* of a future signal *V*(*o*; π, γ, *c*) = *E*[*G*_*t*_|*o*_*t*_ = *o*] is estimated, given the agent's experience.

General Value Functions (GVFs) learn the value specified by three *question parameters*: a *cumulant* *c*, a *policy* π, and a discount γ. The cumulant is the signal of interest being accumulated. The policy describes the behavior the prediction is conditioned on. The discount function describes the temporal horizon over which we wish to make the prediction and how the cumulant is accumulated. Using these three parameters, functions about the agent's subjective experience can be expressed as *returns* $G_{t}^{\pi }=\sum_{k=0}^{\infty }\gamma^{k}C_{t+k+1}$. An agent can then learn a model that estimates the *return*, or *value* of a GVF given its current sensations *via* TD learning (Algorithm 1).

**Algorithm 1:** TD(λ).

1: Initialize eligibility traces *z*∈0^*n*^, weights *w*∈0^*n*^; initialize a step-size 0 < α ≤ 1 and eligibility decay 0 ≤ λ ≤ 1; observe environment *o*
2: Repeat for each observation *o* and cumulant *c*:
3: $\delta \leftarrow c+\gamma w^{⊤}x\left(o_{t+1}\right)-w^{⊤}x\left(o_{t}\right)$
4: For *i* = 1, 2, ⋯ , *n*:
5: *z*_*i*_←*z*_*i*_γλ+*x*_*i*_(*o*_*t*_)
6: *w*_*i*_←*w*_*i*_+α_*i*_δ*z*_*i*_
7: *o*_*t*_←*o*_*t*+1_

## 4. Explanation to Self and Others: How an Agent Interprets Its Own Estimates and Shares Them

If the question parameters of a GVF are explainable, then it is apparent what a prediction is about by looking at the question parameters themselves. The cumulant *c*(*o*_*t*_), policy π(*o*_*t*_, *a*_*t*_), and discount γ(*o*_*t*_, *a*_*t*_, *o*_*t*+1_) describe what a prediction is about in terms of environmental stimuli to the agent. While these parameters do not provide an intuitive explanation in terms of natural language or symbols, the cumulant, discount, and policy *are* inherently interpretable.

While we can determine *what* a prediction is about by examining the question parameters, the question remains if we determine *why* a decision or estimate was made by looking at the model that produced the estimate: e.g., given an observation *o*_*t*_, why is the value *v*(*o*_*t*_)? Whether we can determine why an estimate was made depends on whether function approximation is used.

If a particular GVF is a linear combination of features and weights, it is inherently interpretable (Molnar, 2020; Ghassemi et al., 2021): coefficients *w*_*i*_ are directly multiplied with each input *o*_*i*_, and each coefficient *w*_*i*_ determines how an estimate *v*(*o*_*t*_) was reached by weighting the observations. However, many value functions cannot be characterized by a linear relationship. By using *function approximation* (e.g., Sherstov and Stone, 2005; Mnih et al., 2015; Travnik and Pilarski, 2017) non-linearity is added to the inputs, producing a new vector *x*(*o*_*t*_) with which we linearly combine with the models weights in order to produce an estimate $v\left(o_{t}\right)=w^{⊤}x\left(o_{t}\right)$. The weights w are no longer inherently interpretable since they are learned such that they combine with *x*(*o*_*t*_).

Interpreting the model's weights when using function approximation and making sense of its estimates is challenging, as is the case with non-linear many ML methods. However, an agent may use *post-hoc* interpretation (Molnar, 2020) methods to justify its decisions. In this discussion, we contribute a distinction between two forms of explanation: *introspective explanation*—how an agent analyses and evaluates its own internal signals and decisions; and, *exteroceptive explanation*—how an agent communicates its internal signals to other agents or individuals.

## 5. Introspective Explanation

The problem of introspective explanation within the context of predictive knowledge is then the problem of an agent deciding whether or not an estimate—whether a particular GVF within the agent's model—is sufficient to rely on for decision-making. Prior to rationalizing a decision to external agents and human designers, an agent must commit to a decision itself—an agent must choose an action *a*_*t*_ using the information available to itself *o*_*t*_. In predictive knowledge systems, GVF forecasts are often a key component of this observation *o*_*t*_ (Edwards et al., 2016b; Günther, 2018; Kearney et al., 2021; Ring, 2021; Schlegel et al., 2021). However, not all predictions are created equally.

The accuracy and precision of predictions are dependent on a variety of factors, including the learning methods used to generate the model, the hyperparameters that modulate learning, and the inputs or features available to learn a model from. Two models of the environment can have different numerical values, despite being *about* the same thing. It is important to differentiate between accurate and inaccurate estimates when they are used as inputs for decision-making tasks. Poor inputs can lead to catastrophic errors; moreover, these errors may not be immediately apparent from the agent's (or human designer's) perspective (Kearney et al., 2020).

Looking only at immediate estimates *v*(*o*_*t*_), faults in an agent's own model and GVF estimates are inscrutable. On each time-step the agent is aware of its estimate *v*(*o*_*t*_) and the *bootstrapped* value observed *c*+γ*v*(*o*_*t*+1_). Although error δ can be calculated from these values (line 6, Algorithm 1) to further learn the estimates, error is often not an input in *o*_*t*_ that influences an agent's decision-making. Without additional information, an agent has no means to discern how reliable an estimate in its model is.

### 5.1. Estimating Variance: A Self-Assessment of Certainty

By monitoring the variance of estimates (Sherstan et al., 2018a), an agent can take into account both the model estimates and the certainty in those estimates during decision-making (Sherstan et al., 2016). By evaluating the qualities of its own estimates and learning progress, an agent can develop a sense of confidence: “how much do I trust that my estimates are accurate?” This ability to incorporate *post-hoc* interpretations (Molnar, 2020) of its own model in decision-making, is a form of self-explanation. In this case, an agent chooses an action not only because of predicted values, but the relative certainty in those values.

An estimate from a newly initialized GVF will often be poor, and for good reason: it has not had time to refine its estimates through experience. An agent which examines the certainty of estimates alone cannot differentiate between newly added components of its model, and genuinely poor estimates. It is possibly to use summary statistics of internal learning to capture measurements of learning progress (Kearney et al., 2019; Linke, 2021). By monitoring how much it has learned about its environment, an agent can use this information as an auxiliary reward to shape how it moves about its environment to further learn useful behaviors (Linke, 2021). In this case actions are taken based on not only what is believed to be rewarding, but also what action would enable the most learning.

### 5.2. Unexpected Error: As Self-Assessment of Surprise

Unexpected Demon Error (UDE) is a metric designed to express an agent's “surprise” about a sensation (White et al., 2015). By calculating UDE, an agent can monitor unexpected changes in either the environment or its own physical body (Pilarski and Sherstan, 2016; Günther et al., 2018; Wong, 2021). Neither error encountered while learning, nor error due to random noise in the environment factor into UDE. As regular predictable sources of error are filtered out, the UDE represents salient sources of error that would not be otherwise noticeable to an agent.

### 5.3. Feature Relevance: A Self-Assessment of Explanatory Variables

Learning processes are governed by parameters that dictate how learning occurs. One important parameter is the *step-size* or *learning-rate*. The step-size α determines how much the model's parameters *w* are updated every time-step based on the observed error δ. The value of a GVF's step-size α can be set by a meta-learning process (Günther et al., 2019; Kearney et al., 2019). Not only do these adaptive step-sizes improve learning, they can also provide useful information about the operation of an agent (Günther et al., 2019). Learned step-size values can also detect anomalies in the agent's observation stream, including those that indicate a hardware or sensor failure of the agent (Günther et al., 2020).

Each internal measurement we discussed is a fragment of an agent's inner life that can directly be accessed and monitored by the agent itself. By introspecting and monitoring their own internal signals, an agent can augment their observations: an agent can act on not only its model of the environment, but it's interpretation of how the model was constructed. By building up the private, subjective experience of an agent, explanation is no longer grounded in symbols that are pre-defined by human designers, but is rooted in the agent's own conception of the world. Each internal measurement we discuss is a measurement that depends only on what is available to the agent. In this case, the agent's ability to explain is limited only by the functions of internal and external signals it can monitor. As an agent adds new predictions to learn, interospective measurements can be added to asses each new GVF of the agent's model, without human intervention. Each individual GVF can be learned separately from the others, and thus each GVF that makes up our model can be interpreted independent of the rest. It is not necessary to assess a collection of predictions monolithically, as is the case with many neural-network based world models. By prioritizing agents that introspect, we provide a basis for explanation that begins with the agent explaining to itself.

## 6. Making Introspection Public: Explanation to Others

Having given an agent the ability to explain its beliefs and decisions to itself, we can begin to make the agent's private inner life public: we can make overt the subjective view of the agent. We view exterospective explanation through two lenses: (1) the behavior the agent undertakes, and (2) the estimates the agent learns and produces. In this section, we show that making the internal signals of the agent's inner life external is a viable form of communication and explanation.

### 6.1. Reflexes: Communication *via* Fixed Responses

The simplest way an agent can communicate with its outside world is by taking an action. The simplest action an agent can take is a reflex: an automatic fixed response to some external stimuli. Reflexes are a foundational form of behavior in both complex and simple biological systems (Gormezano and Kehoe, 1975; Jirenhed et al., 2007; Pavlov, 2010). Artificial agents can learn to anticipate signals (Ludvig et al., 2012), and perform reflexively similar to biological systems (Modayil and Sutton, 2014). Reflexes are inherently explainable, because they are deterministic. By reflexively acting, an agent is making external its internal estimate for a given stimuli. While deterministic, differentiating between reflexes and the stochastic behavior of regular decision-making is not always clear. Moreover, when behaviors are not fixed, interpreting the cause for a particular behavior is more challenging: many different stimuli could possibly elicit the same response from the agent.

### 6.2. Predictions: Communication of Inner Estimates

The challenge of interpreting agents based on behavior alone is evident in existing work using GVF-based systems on biomedical devices and assistive devices (Pilarski et al., 2013; Edwards et al., 2016a,b). In these applications, humans interact with machines that build behaviors based on predictions learned from the machine's subjective experience (Pilarski et al., 2013; Sherstan et al., 2015; Edwards et al., 2016a). Applications of predictive knowledge systems where humans and agents collaborate to control a system, have been shown to improved performance over non-adaptive systems an individual controls directly (Edwards et al., 2016b); however, one overriding theme is that human participants have consistently expressed a lack of trust in these systems where internal predictions that led to decisions were not visible to them (c.f., Edwards et al., 2016b). The actions of the agent in the environment are an insufficient form of communication with human collaborators. When the internal predictions made by a system were made visible in their raw form to a human partner, participants were able to both understand and fruitfully make decisions in the shared environment (Edwards et al., 2016b). In a task where a human collaborator was in direct control of a robotic third arm, when predictions of motor torque (a proxy for impact in a workspace) were mapped to vibration on a human collaborator's arm, the user was better able to perform precise positioning tasks (Parker et al., 2019). Similarly, by using audio cues to communicate a machine's internal prediction of the value of objects in a Virtual Reality environment, humans were able to perform better in a foraging task (Pilarski et al., 2019). In a task where an artificial agent had control over some aspects of a bionic limb, external communication of the predictions driving control were needed to enable well-timed collaboration with a jointly acting human (Edwards et al., 2016b). Put simply—humans often find it uncomfortable to collaborate with a learning machine on a task if they do not have a window into the predictions that inform decision making. By making the agent's internal estimates public, humans are able to learn about the agent and build trust through joint experience. Exploring how internal signals can be made public provides a promising frontier for future work exploring joint-action GVF systems, and a ideal setting to put into practice the suggestions presented in this perspective article.

## 7. Discussion

In this article, we explore GVFs within the context of explainable AI. We discuss how the question and answer parameters that make up a GVF can be interpreted by human designers. We emphasize that self-supervised agents benefit from introspection, arguing that introspection is a form of self-explanation. We explore a wide range of collaborative human-agent GVF systems, highlighting how these agents' success is dependent on making the internal, introspective signals public to their human collaborators. Collaborative human-agent systems are more effective when an agent's internal estimates are made public to collaborators, even though such signals are not necessarily in human terms or symbols. Although predictive knowledge systems are a relatively new area of research, they are a promising way of developing self-supervised and explainable AI.

## Data Availability Statement

The original contributions presented in the study are included in the article/supplementary material, further inquiries can be directed to the corresponding author/s.

## Author Contributions

AK led the research and analysis for this manuscript. JG provided the figures. JG and PP supervised this research. PP secured funding for this research. All authors contributed to the writing, editing, and approval of this manuscript for submission.

## Funding

This research was undertaken, in part, thanks to funding from the Canada Research Chairs program, the Canada CIFAR AI Chairs program, the Canada Foundation for Innovation, the Alberta Machine Intelligence Institute, Alberta Innovates, and the Natural Sciences and Engineering Research Council. AK was supported by scholarships and awards from NSERC.

## Conflict of Interest

The authors declare that the research was conducted in the absence of any commercial or financial relationships that could be construed as a potential conflict of interest.

## Publisher's Note

All claims expressed in this article are solely those of the authors and do not necessarily represent those of their affiliated organizations, or those of the publisher, the editors and the reviewers. Any product that may be evaluated in this article, or claim that may be made by its manufacturer, is not guaranteed or endorsed by the publisher.
